# Deep Sequencing of the Vaginal Microbiota of Women with HIV

**DOI:** 10.1371/journal.pone.0012078

**Published:** 2010-08-12

**Authors:** Ruben Hummelen, Andrew D. Fernandes, Jean M. Macklaim, Russell J. Dickson, John Changalucha, Gregory B. Gloor, Gregor Reid

**Affiliations:** 1 Canadian Research & Development Centre for Probiotics, Lawson Health Research Institute, London, Canada; 2 Department of Public Health, Erasmus MC, University Medical Centre Rotterdam, Rotterdam, The Netherlands; 3 Department Applied Mathematics, The University of Western Ontario, London, Canada; 4 Department of Biochemistry, The University of Western Ontario, London, Canada; 5 Mwanza Research Center, National Institute for Medical Research, Mwanza, Tanzania; 6 Department of Microbiology and Immunology and Department of Surgery, The University of Western Ontario, London, Canada; Charité-Universitätsmedizin Berlin, Germany

## Abstract

**Background:**

Women living with HIV and co-infected with bacterial vaginosis (BV) are at higher risk for transmitting HIV to a partner or newborn. It is poorly understood which bacterial communities constitute BV or the normal vaginal microbiota among this population and how the microbiota associated with BV responds to antibiotic treatment.

**Methods and Findings:**

The vaginal microbiota of 132 HIV positive Tanzanian women, including 39 who received metronidazole treatment for BV, were profiled using Illumina to sequence the V6 region of the 16S rRNA gene. Of note, *Gardnerella vaginalis* and *Lactobacillus iners* were detected in each sample constituting core members of the vaginal microbiota. Eight major clusters were detected with relatively uniform microbiota compositions. Two clusters dominated by *L. iners* or *L. crispatus* were strongly associated with a normal microbiota. The *L. crispatus* dominated microbiota were associated with low pH, but when *L. crispatus* was not present, a large fraction of *L. iners* was required to predict a low pH. Four clusters were strongly associated with BV, and were dominated by *Prevotella bivia, Lachnospiraceae*, or a mixture of different species. Metronidazole treatment reduced the microbial diversity and perturbed the BV-associated microbiota, but rarely resulted in the establishment of a lactobacilli-dominated microbiota.

**Conclusions:**

Illumina based microbial profiling enabled high though-put analyses of microbial samples at a high phylogenetic resolution. The vaginal microbiota among women living with HIV in Sub-Saharan Africa constitutes several profiles associated with a normal microbiota or BV. Recurrence of BV frequently constitutes a different BV-associated profile than before antibiotic treatment.

## Introduction

The human vaginal microbiota plays an important role in the maintenance of health of a woman, partner or newborn [1]. Until very recently, the composition and dynamics of these organisms was poorly understood due to limitations of culturing methods. The advent of PCR based techniques and pyrosequencing has made it possible to further examine this complex microbial niche (reviewed in [2]). Herein, we describe the development and utilization of a novel metagenomic approach based on Illumina sequencing to profile the vaginal microbiota. Illumina has fewer errors than 454 sequencing [3] and therefore we hypothesized that it could provide a higher phylogenetic resolution than 454 based approaches [4]. Furthermore, the advantage of Illumina to provide 30-times more reads would enable us to perform in depth sequencing of hundreds of samples in one run at a fraction of the costs, making it an excellent tool for large microbiome studies. We chose to sequence the V6 variable region of the 16S rRNA gene as it was adequate to resolve the majority of the expected organisms in the vaginal microbiota to the genus level [5] and provided resolution for a number of organisms in our samples to species and in some cases to strain level (Gloor *et al.* submitted).

In selecting the population to study, we were struck by the extremely high prevalence of bacterial vaginosis (BV) in women living with HIV in Sub-Saharan Africa [6]–[9]. This condition is generally characterized by a depletion of lactobacilli and over-colonization by a range of anaerobic bacteria [10]. The apparent absence of lactobacilli and their by-product lactic acid increases vaginal pH, thereby disrupting physiological mechanisms that might inactivate or contain the virus [11]–[14]. These factors presumably result in an increased risk of transmission to a newborn child or partner associated with BV [15]. Knowledge of the microbiota profiles among women living with HIV is important as different microorganisms may be indicative of distinct risk-profiles in the transmission of HIV and severity of BV. Another consideration for selecting this study population was that BV appears to be more recalcitrant to antibiotic treatment in HIV patients and treatment is ineffective in reducing HIV-shedding from the vaginal tract [6], [16]. Assessing the response of the BV-associated microbiota to antibiotic treatment may yield further insight in factors contributing to the failure of treatment. Therefore we sought to define (1) the microbial communities of women living with HIV, (2) their association with the vaginal pH and (3) their relation with the Amsel criteria and Nugent scoring system commonly used to diagnose BV [17], [18].

## Results

A total of 272 samples were sequenced using a single lane of a paired-end Illumina run resulting in >18 million reads of 76 nucleotides. Of these >12 million bar-coded V6 16S rRNA sequences were identified that completely covered the V6 region and that had intact barcodes and primer sequences (see Gloor et al, submitted, for a complete description of the workflow used to extract these sequences). Individual samples were covered with a median of 40,931 reads (range 7,018–129,054). The Chao1 measure of biodiversity estimation was the most conservative estimate and predicted that the reads covered >90% of the expected diversity in 235 of 272 samples (Table 1). After clustering in operational taxonomic units (OTUs) based on a >95% sequence similarity cut-off, which permitted up to 3 substitutions per V6 sequence, a total of 60 OTUs representing phylogenetic groups were identified (Tables S1, S2, Figures S1, S2). Of those, 30 were assigned to specific species based on sequence identity to a well-annotated organism and 18 to a genus. Strikingly, within some OTUs distinct strains could be separated. For example, within OTU 1 the sequences could be annotated as *Gardnerella vaginalis* 409-05, *G. vaginalis* NML060420, and as two unique uncultured *G. vaginalis* sequences. All four of these sequences differed from each other by a single diagnostic nucleotide.

**Table 1:** Coverage estimates.

Method	N>0.9	N>0.95
Rarefaction	272	255
Ace	241	169
Chao1	235	181

The number of samples in which >90% and 95% of the expected diversity was sampled using rarefaction, Ace and Chao 1 estimates [34], [35]. Analyses were performed using the VEGAN package for biodiversity analyses [36].

### *L. iners* and *G. vaginalis* constitute core members of the vaginal microbiota

**Figure 1:**
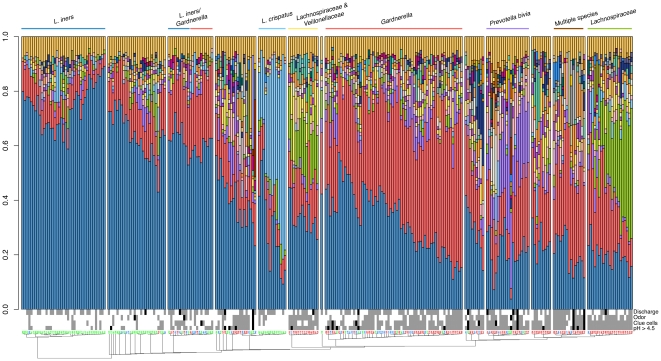
Relative abundance of taxa across samples. The composition of the microbiota was visualized by bar-plots. Samples were clustered by the similarity of the relative organism abundance and their similarity was visualized using a neighbor-joining tree. The major clusters (>10 samples) are named after a taxa with a relative even abundance across samples in the cluster. The sample numbers are coloured according to Nugent categories with BV =  red, intermediate  =  green, normal  =  blue. Amsel criteria are shown for each sample with present  =  grey, absent  =  white and missing data  =  black. The complete legend is given in figure S2.

Figure 1 shows the composition of the microbiota clustered by the similarity of the relative abundance of the organisms. We found that 207 of the 272 samples could be grouped into 8 major clusters with relatively uniform microbiota compositions. Across all clusters, *G. vaginalis* and *Lactobacillus iners* were detected in each sample, constituting core members of the microbiota. Two major clusters were associated with a normal vaginal microbiota, one dominated by *L. iners* and one dominated by *L. crispatus*. Four clusters were strongly associated with BV, and were dominated by *Prevotella bivia* or *Lachnospiraceae*, or comprised a mixture of different species. The remaining clusters were notable in that they contained normal, intermediate and BV samples and were composed largely of *G. vaginalis* and *L. iners* and a mixture of other species.

### The vaginal microbiota and pH

**Figure 2:**
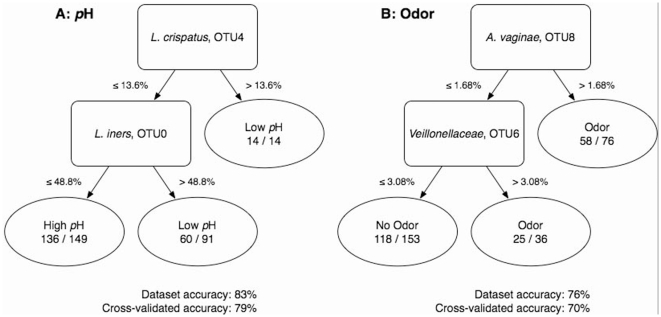
Association of vaginal pH and odor with members of the vaginal microbiota. The flow chart explaining the relationship between microbiota and diagnostic criteria were generated using data mining techniques (Materials and Methods). To obtain the prediction about a clinical sample, follow the arrows like a flow chart from the organisms (rectangular boxes) to the prediction (ovals). Each decision in the tree is split on the proportion of the organism above; thus, if a clinical sample has proportions less than 13.6% *L. crispatus* and less than 48.8% of *L. iners* it is predicted that the sample is taken from a vaginal environment with a pH≥4.5. The accuracy of the decision tree is reported for the entire dataset and for 10-folds cross validation, which is an attempt to estimate the accuracy of the decision tree on new clinical samples.

Figure 1 shows that all 13 samples within the *L. crispatus* cluster, but only 29 of 39 samples in the *L. iners* cluster had a pH≤4.5 (Table 2). We used two independent methods to examine the strength of this association. Table 2 shows that the proportion of *L. iners* alone was only weakly associated with the vaginal pH. The strength of this association increased when the proportion of *L. crispatus* was added to the model. Figure 2A shows that *L. crispatus* is associated with a low pH, but if *L. crispatus* is not present, a large fraction of *L. iners* is required to predict a low pH. However, lack of both *L. iners* and *L. crispatus* is strongly predictive of high pH.

**Table 2:** Correlation Amsel and Nugent score with vaginal microbiota clusters.

Dominant organisms	n	*p*H		Clue cells		Odor		Nugent score		
		≤4.5	>4.5	no	yes	no	yes	0–3	4–6	7–10
*L. iners*	39	29	10	33	6	35	4	38	1	0
*L. iners & G. vaginalis*	21	9	12	11	9	16	4	7	8	6
*L. crispatus*	13	13	0	11	2	10	3	13	0	0
*Lachnospiriceae & Veillonellaceae*	14	1	12	4	10	1	13	0	1	13
*G. vaginalis*	64	9	50	16	46	35	29	9	19	36
*Prevotella bivia*	20	0	18	4	14	7	11	3	4	13
*Multiple species*	15	2	10	2	11	3	10	0	3	12
*Lachnospiraceae*	21	0	21	0	21	5	16	0	1	20

This table summarizes the number of samples for each cluster (corresponding to Figure 1) with a specific Nugent or Amsel diagnostic characteristic. Categories do not always count up due to missing data (Figure 1).

### The vaginal microbiota and the Amsel criteria

Table 3 shows that the various components of the Amsel criteria vary in their strength of association with the vaginal microbiota. In particular, the presence of a milk-like discharge did not appear to have any correlation with the resident microbiota and was a confounding factor within the set of four Amsel criteria (Tables 3, S3). The presence of odor was moderately associated with the vaginal microbiota. Of note, two *Lachnospiriceae* clusters, and the multiple species cluster appeared to be most strongly associated with the presence of an amine odor (Figure 1 and Table 2). Figure 2B show that *Atopobium vaginae* and *Veillonellaceae* were most clearly associated with odor across clusters.

**Table 3:** Correlation proportion of lactobacilli with Amsel criteria and Nugent score.

Variable	Proportion	Pairs	Association^1^	*p*	Strength^2^
pH	*L. iners*	254	−0.2469	0.0000	83
pH	*L. iners* & *L. crispatus*	254	−0.2311	0.0000	35
Nugent score	*L. iners* & *L. crispatus*	272	−0.2423	0.0000	34
Amsel score	*L. iners* & *L, crispatus*	242	−0.1531	0.0012	31
Odor	*L. iners & L. crispatus*	265	−0.1473	0.0034	231
Clue cells	*L. iners* & *L. crispatus*	262	−0.1927	0.0001	55
Discharge	*L. iners* & *L. crispatus*	259	0.0514	0.3131	418

1 Kendall rank correlation coefficient and p-value.

2 Strength of the association is indicated by estimating the number of subjects that would be required to show the correlation between the variables with ≥95% confidence.

### Impact of Metronidazole treatment

**Figure 3:**
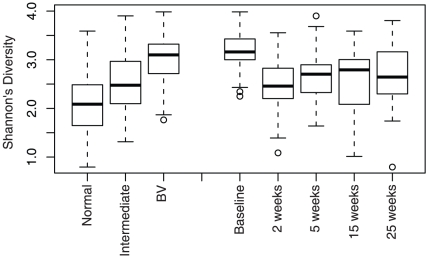
Diversity of the vaginal microbiota and the impact of antibiotics. The species diversity was measured by calculating Shannon's Diversity index [31]. The data was partitioned either by Nugent category [17], or by treatment time-point. Metronidazole treatment (week 0) reduced the diversity at two weeks but a slightly higher diversity was observed at later follow-up time-points indicating a return to a more diverse microbiota.

**Figure 4:**
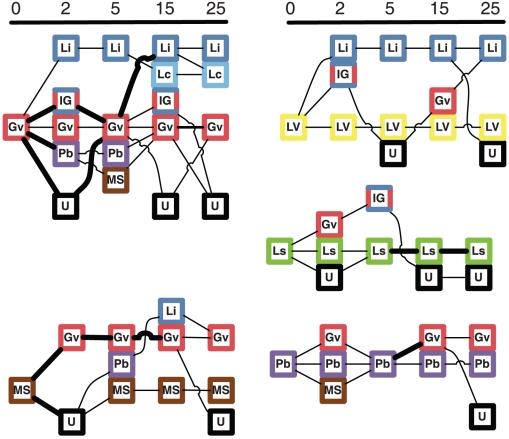
Changes in microbiota profile after antibiotic intervention. The network depicts the profile of the vaginal microbiota before (time  = 0) and after metronidazole intervention. A thin line represents one subject while a thick line represents two. Only women were included with a complete follow-up and when a microbiota was present similar to the major profiles at baseline (Figure 1). Gv indicates *G. vaginalis*, MS =  Multiple species, LV =  *Lachnospiraceae* & *Veillonellaceae*, Ls =  *Lachnospiraceae*, Pb =  *P. bivia* and U =  minor clusters.

**Figure 5:**
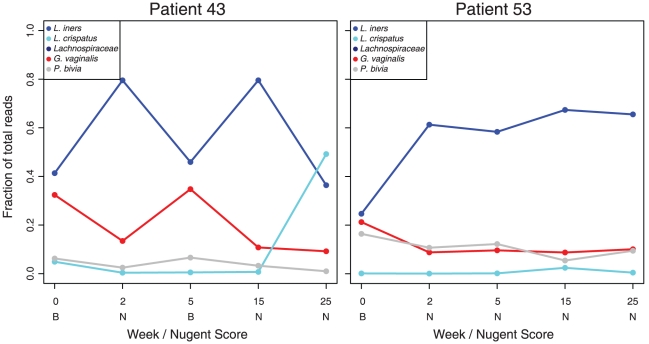
Dynamics of the vaginal microbiota after antibiotic intervention. The line graphs depict the changes in relative abundance for a select number of species after metronidazole intervention (week 0). After two weeks both patients are cleared of BV along with an increase in the relative abundance of *L. iners* at two weeks. However patient 43 experiences a recurrence and receives another course of metronidazole at 5 weeks. Of note, after an *L. crispatus* profile is established, BV does not recur in this subject.

Of the 132 women enrolled at baseline, 47 had a normal microbiota, 22 intermediate and 67 had BV as defined by the Nugent score [17]. The microbiota of BV patients were composed a larger diversity of taxa than normal subjects (Figure 3). All subjects with BV were treated with metronidazole and 39 subsequently returned for follow-up visits. After 2 weeks, 57% of these patients still had BV. Treatment with metronidazole reduced the diversity at two weeks but the diversity increased slightly at later follow-up time-points indicating a tendency to return to a more diverse microbiota (Figure 3). Metronidazole frequently caused a shift from one BV-associated microbiota profile to another, but rarely resulted in a lactobacilli-dominated microbiota (Figure 4). *L. iners* was the only organism with an increase in relative abundance (from a median of 25% to 46%) after metronidazole intervention (p = <0.0001, Wilcoxon rank test). The effect of metronidazole on two patients is presented in Figure 5, to illustrate the dynamics of microbiota changes with antibiotic intervention.

## Discussion

To our knowledge, we report the first successful attempt to nearly completely sample the diversity of the vaginal microbiota. Compared to 454 sequencing, the Illumina method provided 30-fold more reads with a 10-fold reduction in costs, enabling hundreds of samples to be processed in one run. This method could be adapted to sequence the V3 or V5 region suitable for uncovering the intestinal microbiota [5]. The reduced costs of microbial profiling ($10/sample) might enable the use of this technology in a diagnostic setting and allow large studies to be carried our on the human microbiota.

Based on similarity of the relative abundance, we have demonstrated in an HIV population of African women, that there are several microbiota profiles associated with BV, and two with a normal, healthy status. For BV cases, there was a dominant abundance of *P. bivia* or members of the order *Clostridiales* and family *Lachnospiraceae*. Interestingly, in a culture based study of African women, *P. bivia* was also found to be the dominant organism [7], unlike Caucasian and black women from North America, in whom *Atopobium vaginae* and genera of the order *Clostridiales,* such as *Megasphaera* sp., were dominant [10], [19]. A recent 454 pyrosequencing study of a healthy cohort of American women including African Americans, showed the *Prevotella* genus to be the most abundant in one of five clusters [20]. *Prevotella bivia* is a well known pathogen that has been reported to invade epithelial cells, cause inflammatory responses, endometritis, pelvic inflammatory disease, and peri-rectal abscesses [21]. Its presence in the vagina of HIV patients warrants efforts to treat and eradicate it; however, this was not achieved by the use of metronidazole in the present study.

The common finding of *Clostridiales* organisms, presumably originating from the gut, indicates an ability to adapt easily to the vaginal environment. The two clusters in which *Lachnospiraceae* were heavily represented, were associated with the presence of an amine odor. Although many cases of BV can be asymptomatic, odor is a major reason for women deciding to seek treatment. It is unpleasant and adversely affects quality of life [22]. The association of *Clostridialis* organisms and *A. vaginae* with odor warrants the evaluation of interventions specifically directed against these organisms.

The presence of *L. crispatus* or a high abundance of *L. iners* was clearly associated with a healthy pH<4.5. The fact that *L. iners* was detected in all the vaginal samples, along with *G. vaginalis*, indicates they have some core functions or at least a well developed means to persist in the fluctuating environment of the vagina. It would be worthwhile examining a larger sample pool of women colonized by *L. crispatus*, to verify what appears to be an important role in lowering the vaginal pH. The ability of *L. crispatus*, unlike *L. iners*, to produce hydrogen peroxide may also be a factor in maintenance of health [23]. The low prevalence of *L. crispatus* and the virtual absence of *L. jensenii* relative to non-African populations [10], [19], [23]–[25] might in part explain the high occurrence and low cure rates of BV among this cohort of Tanzanian women with HIV.

Treatment with metronidazole was associated with a shift in microbial profiles without necessarily a recurrence of the same profile, and poor efficacy at establishing a lactobacilli-dominant microbiota. The treatment caused a shift in abundance of *L. iners*, but did not induce a recovery of *L. crispatus*. This is interesting for several reasons. The emergence of BV causes a displacement or killing of *L. crispatus*, while it does not eradicate *L. iners*. The genome of the latter contains exogenously acquired elements that appear to aid persistence and survival (Macklaim *et al.* manuscript submitted), but how they achieve this when confronted by biofilms of BV pathogens, remains to be determined. If such mechanisms are identified, it may be possible to try and up-regulate them in probiotic strains such as *L. crispatus* CTV05, *L. rhamnosus* GR-1 or *L. reuteri* RC-14. By surviving BV and antibiotic treatment, *L. iners* may be able to proliferate (as shown by its increased abundance following metronidazole treatment) and create an environment conducive to later re-colonization by *L. crispatus* via the rectum and perineum. This remains to be verified.

In summary, we report a novel, inexpensive, highly practical technique for profiling the human microbiota at a high phylogenetic resolution. The study identified 60 OTUs in the vagina of African women with HIV and *L. iners* and *G. vaginalis* were found to be core members of the vaginal microbiota. Four bacterial clusters were associated with BV, and two clusters with a normal vaginal microbiota. *L. crispatus* was more strongly associated with a low vaginal pH than *L. iners*, suggesting a different ecological function. Treatment failed to eradicate BV and only transiently reduced the diversity of the BV-associated microbiota.

## Materials and Methods

### Clinical study and sample collection

Women visiting the HIV clinic of Sekou-Toure Regional Hospital in Mwanza, Tanzania, were invited to participate in the study. Subjects were included when: their HIV infected status was confirmed, they had not yet initiated antiretroviral treatment, and they were between 18 and 45 years old. Participants were not included when pregnant, lactating, menstruating at time of screening, hypersensitive to metronidazole, had *Trichomoniasis* detected by saline microscopy, had budding yeast indicating *Candida* colonisation seen on Gram stain or had cervical inflammation noted during gynaecologic examination. The medical ethical review committee of Erasmus University Medical Centre, The Netherlands, and the medical research coordinating committee of the National Institute for Medical Research, Tanzania, approved the study design and protocol. Subjects were informed of the purpose of the study and gave their signed informed consent before participation. The study was registered at clinical trials.gov NCT00536848.

A total of 132 women were screened of whom a sub-group of women (n = 39) diagnosed with BV (Nugent score ≥7) were treated with metronidazole (400mg orally, twice daily for 10 days) (Aventis), and followed at 2, 5, 15 and 25 weeks. If symptoms and signs of BV in addition to a Nugent score of ≥7 were present at any follow-up visit from week 5, subjects were again prescribed metronidazole. At each visit, vaginal samples were collected for Gram staining, pH, saline microscopy and KOH preparation. Samples for DNA extraction and PCR were obtained by brushing a polyester swab against the mid-vaginal wall after insertion of a non-lubricated speculum. The swabs were placed in a cryovial and stored at −20°C until analysis. BV was diagnosed at each visit by rating a gram stained smear by Nugent scoring [17] and Amsel's criteria [18]. A Nugent score of 1–3 was defined as normal, 4–6 as intermediate and 7–10 as BV. Also, the presence of trichomoniasis was diagnosed by saline microscopy and the presence of budding yeast by Gram stain to indicate *Candida* colonisation.

### PCR amplification

The primers L-V6 (5′-CAACGCGARGAACCTTACC-3′) and R-V6 (5′-ACAACACGAGCTGACGAC-3′) were chosen to amplify the V6 hypervariable region of the 16S rRNA gene. The primers were tested *in silico* using a custom made database including all entries in the RDP with taxonomic information and longer than 1400 nucleotides. Of all those sequences the left primer matched over 96% and the right primer over 98% of the sequences in the dataset. The taxa *Sneathia*, *Leptotrichia*, *Ureaplasma* and *Mycoplasma* could only be amplified if up to 4 mismatches were allowed outside the first 5 nucleotides of the left primer, and therefore we suggest that our data are biased against these taxa. We developed different barcodes of 3–6 nucleotides long, to be attached to the 5′ end of the primer that would give a relatively even mixture of the 4 nucleotides at the first several positions. The barcodes were between 3 and 6 nucleotides long to reduce the likelihood that adjacent spots on the Illumina solid support would be scored as one spot during the sequencing of the amplification primers. For the left primer we developed 18 different barcodes (5′-3′CATGCG, GCAGT, TACGT, GACTGT, CGTCGA, GTCGC, ACGTA, CACTAC, TGAC, AGTA, ATGA, TGCA, ACT, TCG, GTA, CTA, TGA, GCTA) and for the right primer 16 different barcodes (5′-3′ CGCATG, ACTGC, AGCTA, ACAGTC, TCGACG, GCGAC, TACGT, GTAGTG, GTCA, TACT, TCAT, TGCA, AGT, CGA, TAC, TAG). A-priori the primers and DNA extraction procedures were tested using the amplification conditions described below on *L. iners*, *L. rhamnosus*, *A. vaginalis* and *G. vaginalis.*

### DNA extraction and amplification

Swabs were vigorously agitated in 1 mL of PBS (pH 7.5) to dislodge cells after which the DNA was extracted using InstaGene Matrix (Biorad #732-6030) according to the protocol of the manufacturer. After extraction, 10 µl of the Instagene supernatant was used as template for a 50 µl PCR reaction. The following PCR reaction conditions were used: 5 units Taq platinum: 1.7mM MgCl, 210 mM dNTPs and 640 nM of each primer. A touch-down protocol was employed with: initial denaturation 94°C for 2 min; denaturation 94°C; annealing starting at 61°C and dropping with 1°C over 10 cycles with the remaining 15 cycles at 51°C; extension at 72°C; all for 45 seconds and a final elongation step for 2 min. A negative control including all ingredients but with water instead of DNA template, and a positive control with a lower limit of detectable DNA, were performed alongside all test reactions. PCR-products were used when the negative control was free of PCR product and the positive control amplified. A constant volume aliquot of each amplification product was run on a 1.5% agarose gel to determine the approximate amount of product. The amount of product was scored on a 4 point scale and, based on this scale, between 2 and 40 µl of the PCR products were mixed together to give the final sample sent for Illumina sequencing at The Next-Generation Sequencing Facility in The Centre for Applied Genomics at the Hospital for Sick Children in Toronto. The library was prepared without further size selection. All new sequencing data have been deposited in GenBank under accession numbers: HM585291-HM585350.

### Taxonomic classification of sequence reads

The number of reads and the short nature of the reads precluded the use of the data analysis pipelines currently in use for microbial community profiling by the 454 pyrosequencing method. We developed a data analysis pipeline and programs in-house (Gloor et al submitted). A shell script plus the Perl and R scripts able to run on OS X to recapitulate the analyses are available from the authors. Sequences present at >1% abundance in any sample were clustered by similarity to a seed sequence at 95% identity using UCLUST [26]. All clustering methods present a trade-off between sensitivity and specificity. The 95% identity cut-off was chosen because it was the best compromise between clustering of sequences derived from PCR-based sequencing errors and true taxonomic relatedness (See figure S1 for full justification of clustering cut-off). The most abundant sequence of each OTU was chosen as seed sequence. Each resultant seed sequence was first compared using mega BLAST against non-redundant bacterial nucleotide sequences in the NCBI database while excluding environmental samples. The best scoring hit was selected to represent the seed sequence if it displayed 100% identity and coverage. If multiple hits fulfilled these criteria, a common higher taxonomic rank was assigned. When no hits were present at >90% identity, uncultured samples were included in the search (Tables S1, S2, Dataset S1). Preference was given in taxonomic assignment to sequences derived from fully sequenced bacterial genomes, then to sequences derived from characterized organisms and finally to environmental isolates.

### Association analyses

Kendall′s Tau was used to test nonparametric rank association between lactobacilli fractions, the Nugent and Amsel criteria primarily for its simple interpretation [27], [28]. The magnitude of association between the interval-scale *Lactobacillus* population fraction and ordinal-scale diagnostic criterion was quantified by a novel technique based on information theory [29]. To ensure invariance to distribution, population fractions were converted to ranks. Sampling variance was preserved by noting that the joint percentile distribution of all variables follows a Dirichlet distribution [30]. The Kullback-Leibler divergence of the uniform density to the empirical density was used to estimate the expected accuracy for distinguishing populations. To facilitate interpretation, the information divergence was converted to an estimate of the number of patient evaluations required to determine with ≥95% confidence whether or not those patients came from a population where no association was present. The more patients needed, the smaller the association between variables.

### Hierarchical clustering among samples

Samples were clustered for similarity of relative abundance by calculating a generalized angle between proportions, as given by their Euclidian inner product [31]. Such a distance is equivalent to minimum Fisher information connecting two points. From a biological perspective, this angle can be loosely interpreted to measure how much a mixture of the two populations would have in common with each of its components. Small angles imply that two samples are virtually identical, while large angle imply that samples are maximally distinct.

### Diversity estimations

Diversity was calculated using Shannon's Diversity Index which calculates the entropy or variability of the system [32]. It is calculated as follows: *H_s_ = −∑n_i_* ln *n_i_* where H is the entropy, and we are summing the species proportions multiplied by the log of those fractions. The diversity index has a minimum value when there is only one species and a maximum when all species are equally and identically distributed.

### Decision trees

Decision trees were generated using the j48 implementation of the C4.5 decision tree algorithm from the Weka machine learning workbench [33]. The C4.5 algorithm attempts to create the most effective decision tree to create accurate predictions by using information theory. At each step in the process, the program splits the dataset on an attribute (eg. the relative abundance of an organism) to maximize the information gained about an important predicted variable (eg. the likelihood of a pH>4.5); the split is then entered into the decision tree like an entry in a flowchart and the process is repeated until no more information can be gained. The decision trees were created using default C4.5 parameters and were pruned to prevent over-fitting the training set. The C4.5 decision trees were validated using 10-folds cross validation.

## Supporting Information

Figure S1 Clustering cut-off neighbor-joining tree. A neighbor-joining tree was constructed from pairwise Levenshtein distances of the identical sequence units (ISU) sequences present at greater than or equal to 1% or more in any sample. The tree shows the ISU sequences that were clustered at sequence identity cut-offs of 92% (green) and 95% (red). These cut-offs correspond to allowing 5 and 3 sequence mismatches in the V6 rRNA sequence. The very large number of sequence reads, coupled with the high abundance of *L. iners* and *G. vaginalis* and several other species resulted in a significant proportion of reads having > 2 mismatches because of PCR amplification induced errors (Gloor et al, submitted). This can be seen clearly in the *L. iners* cluster at 1 o'clock. The seed sequence is ISU 0, and ISU 344 differs from ISU 0 by 3 PCR-induced errors. Clustering at 97% identity would result in false positive identification of a new species, similar to *L. iners*. Thus, the 95% identity clustering cut-off captured all the PCR errors into the proper taxonomy-based clusters. Clustering at 92% identity, shown by the nodes in green, brings obviously distinct taxonomic sequences into the same OTU, and would result in false-negative assignment of too few taxa.
(0.27 MB PDF)

Figure S2 Complete legend barplot relative abundance of taxa across samples. This legend for figure 1 includes all 60 OTUs detected in the samples for identification in the graph.
(3.04 MB PDF)

Table S1 Taxonomic classification of seed sequence OTUs. The table lists the taxonomic classification of the most abundant sequence within each OTU (seed sequence). Each seed sequence was first compared using mega-BLAST against non-redundant bacterial nucleotide sequences excluding environmental samples in the NCBI data-base. The best scoring hit was selected to represent the seed sequence if it displayed 100% identity and coverage. If multiple hits fulfilled these criteria, a common higher taxonomic rank was assigned. When no hits were present at >90% identity, uncultured samples were included in the search. Preference was given in taxonomic assignment to sequences derived from fully sequenced bacterial genomes, then to sequences derived from characterized organisms and finally to environmental isolates.
(0.04 MB XLS)

Table S2 Full length 16S rRNA seed sequence of OTUs. This table lists the full length sequence of the most abundant read within each OTU (seed sequence) after clustering at >95% sequence similarity.
(0.03 MB XLS)

Table S3 Association prevalence of organisms with Amsel criteria and Nugent score. This table summarizes the presence of species at >1% abundance and having BV according to the Nugent score or the Amsel criteria. It demonstrates that the prevalence of species has a limited predictive ability and for having BV.
(0.07 MB DOC)

Dataset S1 Subject diagnostic information and vaginal microbiota composition. This dataset contains the number of reads for each OTU in each sample. Variables are Follow-up, 0 (baseline, no- follow-up), 1 (baseline, followed-up), 2 (follow-up 2 weeks), 3 (follow-up 5 weeks), 4 (follow-up 15 weeks), 5 (follow-up 25 weeks); Subject ID; Sample ID; Nugent score (0–10); Amsel discharge (y/n); Amsel odor (y/n); Amsel clue cells (y/n); pH; Amsel score (0–4); Total number of reads; Number of reads for each OTU.
(0.18 MB XLS)
