# Supplementary figures and images for: Deep Sequencing of the Vaginal Microbiota of Women with HIV
Source: PLoS One. 2010 Aug 12;5(8):e12078. doi: 10.1371/journal.pone.0012078 (PMC2920804; doi:10.1371/journal.pone.0012078)

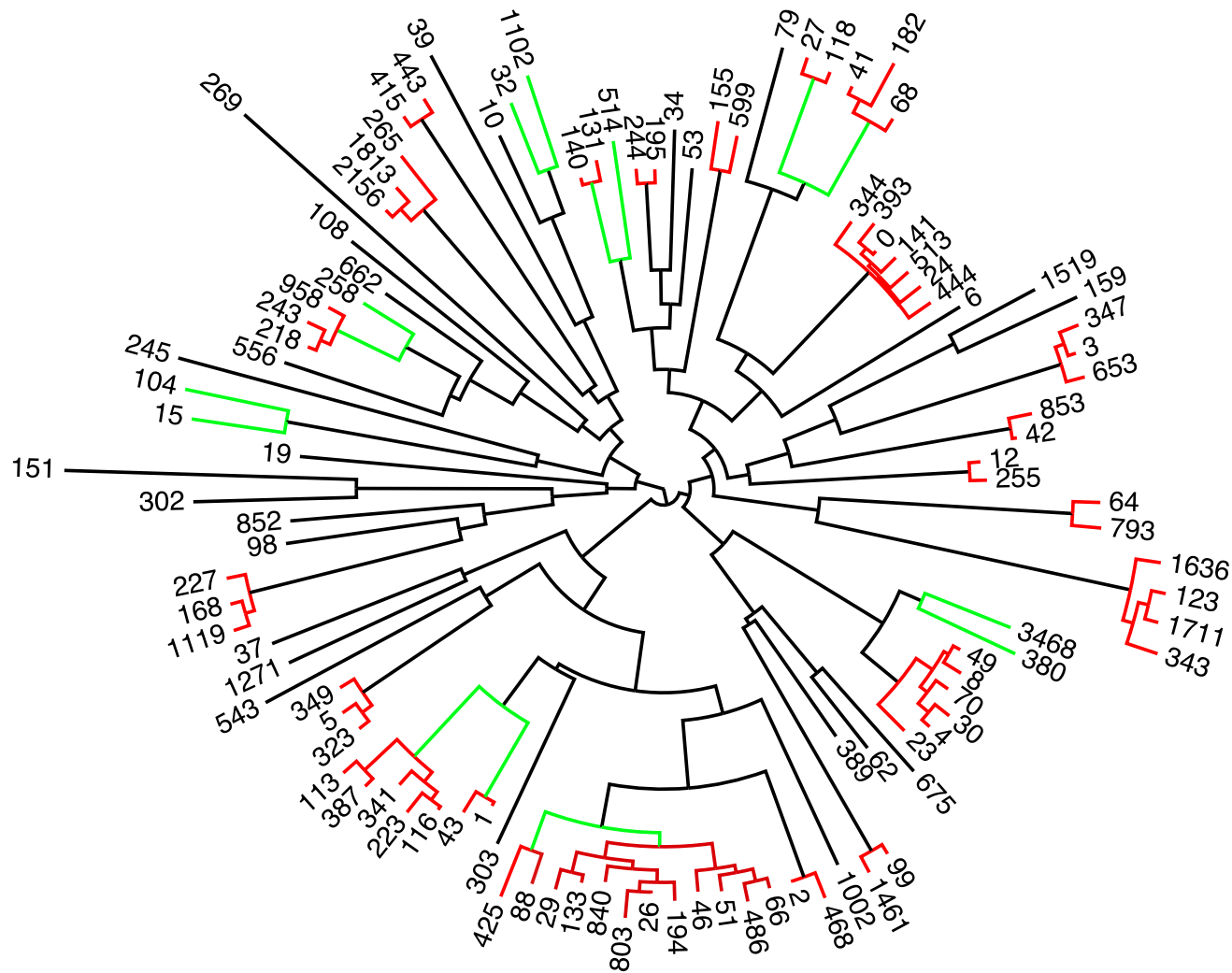

Supplement: Figure S1 — Clustering cut-off neighbor-joining tree. A neighbor-joining tree was constructed from pairwise Levenshtein distances of the identical sequence units (ISU) sequences present at greater than or equal to 1% or more in any sample. The tree shows the ISU sequences that were clustered at sequence identity cut-offs of 92% (green) and 95% (red). These cut-offs correspond to allowing 5 and 3 sequence mismatches in the V6 rRNA sequence. The very large number of sequence reads, coupled with the high abundance of L. iners and G. vaginalis and several other species resulted in a significant proportion of reads having > 2 mismatches because of PCR amplification induced errors (Gloor et al, submitted). This can be seen clearly in the L. iners cluster at 1 o'clock. The seed sequence is ISU 0, and ISU 344 differs from ISU 0 by 3 PCR-induced errors. Clustering at 97% identity would result in false positive identification of a new species, similar to L. iners. Thus, the 95% identity clustering cut-off captured all the PCR errors into the proper taxonomy-based clusters. Clustering at 92% identity, shown by the nodes in green, brings obviously distinct taxonomic sequences into the same OTU, and would result in false-negative assignment of too few taxa. (0.27 MB PDF) [file pone.0012078.s001.pdf]
