# Supplementary material for: Deep Sequencing of the Vaginal Microbiota of Women with HIV
Source: PLoS One. 2010 Aug 12;5(8):e12078. doi: 10.1371/journal.pone.0012078 (PMC2920804; doi:10.1371/journal.pone.0012078)
Supplement: Figure S2 — Complete legend barplot relative abundance of taxa across samples. This legend for figure 1 includes all 60 OTUs detected in the samples for identification in the graph. (3.04 MB PDF) [file pone.0012078.s002.pdf]

- Rare OTUs
- Corynebacterium ammoniagenes, OTU 102
- Uncultured clone (97%), OTU 76
- Uncultured Porphyromonas, OTU 70
- Novel, OTU 68
- Haemophilus, OTU 65
- Bifidobacterium bifidum, OTU 64
- Campylobacter, OTU 63
- Bifidobacterium saeculare (97%), OTU 61
- Gammaproteobacteria, OTU 60
- Mycoplasma pirum (97%), OTU 59
- Uncultured Methylocaldum tepidum, OTU 57
- Fusobacterium nucleatum, OTU 56
- Uncultured Lachnospiraceae (85%), OTU 52
- Peptoniphilus lacrimalis, OTU 50
- Uncultured clone (88%), OTU 48
- Uncultured clone, OTU 47
- Gardnerella vaginalis (97%), OTU 46
- Prevotella, OTU 45
- Mobiluncus curtisii, OTU 44
- Uncultured Sneathia (92%), OTU 42
- Uncultured Fusobacterium (98%), OTU 41
- Arcanobacterium (94%), OTU 39
- Uncultured Prevotella (95%), OTU 38
- Uncultured TM7 division, OTU 37
- Actinomyces turicensis, OTU 36
- Enterobacteriales, OTU 35
- Gemella, OTU 34
- Pasteurellaceae, OTU 33
- Uncultured Prevotella (98%), OTU 32
- Lactobacillus jensenii, OTU 31
- Enterobacteriaceae, OTU 30
- Anaerococcus tetradius, OTU 29
- Lactobacillus, OTU 28
- Gemella, OTU 27
- Porphyromonas, OTU 26
- Clostridiales, OTU 25
- Actinomycetaceae, OTU 24
- Clostridiales BVAB-3, OTU 23
- Peptostreptococcus anaerobius, OTU 22
- Streptococcus, OTU 21
- Streptococcus, OTU 20
- Streptococcus, OTU 19
- Prevotella melaninogenica, OTU 18
- Uncultured Prevotella (90%), OTU 17
- Dialister micraerophilus, OTU 16
- Atopobium rimae, OTU 15
- Veillonella montpellierensis, OTU 14
- Lactobacillaceae, OTU 13
- Dialister propionicifaciens, OTU 12
- Uncultured Peptococcaceae (87%), OTU 11
- Prevotella amniotica, OTU 10
- Prevotella timonensis, OTU 9
- Atopobium vaginae, OTU 8
- Sneathia, OTU 7
- Uncultured Veillonellaceae, OTU 6
- Leptotrichia amnionii, OTU 5
- Lactobacillus crispatus, OTU 4
- Uncultured Lachnospiraceae, OTU 3
- Prevotella bivia, OTU 2
- Gardnerella vaginalis, OTU 1
- Lactobacillus iners, OTU 0
