# Supplementary material for: Deep Sequencing of the Vaginal Microbiota of Women with HIV
Source: PLoS One. 2010 Aug 12;5(8):e12078. doi: 10.1371/journal.pone.0012078 (PMC2920804; doi:10.1371/journal.pone.0012078)
Supplement: Table S3 — Association prevalence of organisms with Amsel criteria and Nugent score. This table summarizes the presence of species at >1% abundance and having BV according to the Nugent score or the Amsel criteria. It demonstrates that the prevalence of species has a limited predictive ability and for having BV. (0.07 MB DOC) [file pone.0012078.s005.doc]

**Table S3**. **Number of subjects with taxa found at** >**1% of total microbiota**

| Taxa^1^ | *Nugent score* | | |  | *Amsel criteria* | |  |
| --- | --- | --- | --- | --- | --- | --- | --- |
|  | 1-3  (n=47)  % (n) | 4-6  (n=22)  % (n) | 7-10  (n=67) % (n) | *P*^2^ | 1-2  (n=51)  % (n) | 3-4  (n=75)  % (n) | *P^2^* |
| *Lactobacillus iners^3^* | 85 (40) | 70 (14) | 17 (11) | <0.001 | 77 (39) | 32 (24) | <0.001 |
| *Garnerella* *vaginalis*^3^ | 21 (10) | 55 (11) | 66 (43) | <0.001 | 31 (16) | 60 (45) | 0.002 |
| *Prevotella bivia* | 45 (21) | 50 (11) | 84 (53) | <0.001 | 37 (19) | 80 (60) | <0.001 |
| Uncultured *Lachnospiraceae* | 53 (25) | 36 (8) | 73 (46) | 0.006 | 59 (30) | 59 (44) | 1.0 |
| *Lactobacillus crispatus* | 64 (30) | 46 (10) | 33 (21) | 0.004 | 55 (28) | 43 (32) | 0.2 |
| *Leptotrichia amnionii* | 55 (26) | 46 (15) | 84 (53) | 0.003 | 63 (32) | 75 (56) | 0.2 |
| Uncultured *Veillonellaceae* | 49 (23) | 55 (12) | 89 (56) | <0.001 | 59 (30) | 75 (56) | 0.06 |
| *Snaethia* | 30 (14) | 32 (7) | 76 (48) | <0.001 | 33 (17) | 64 (48) | 0.001 |
| *Atopobium vaginae* | 15 (7) | 32 (7) | 86 (54) | <0.001 | 26 (13) | 67 (50) | <0.001 |
| *Prevotella timonensis* | 38 (18) | 46 (10) | 95 (60) | <0.001 | 47 (24) | 77 (58) | <0.001 |
| *Prevotella amniotica* | 13 (6) | 23 (5) | 52 (33) | <0.001 | 20 (10) | 39 (29) | 0.02 |
| Uncultured *Peptococcaceae* | 30 (14) | 46 (10) | 52 (33) | 0.06 | 39 (20) | 48 (35) | 0.4 |
| *Dialister propionicifaciens* | 9 (4) | 18 (4) | 51 (32) | <0.001 | 12 (8) | 41 (31) | <0.001 |
| *Lactobacillaceae* | 4 (2) | 23 (5) | 30 (19) | 0.004 | 10 (5) | 27 (20) | 0.02 |
| *Veillonella montpellierensis* | 11 (5) | 18 (4) | 16 (10) | 0.6 | 6 (3) | 17 (13) | 0.06 |
| *Atopobium rimae* | 0 | 0 | 33 (21) | <0.001 | 4 (2) | 24 (18) | 0.002 |
| *Dialister micraerophilus* | 0 | 5 (1) | 18 (11) | 0.001 | 4 (2) | 12 (9) | 0.2 |
| Unculterd *Prevotella* | 0 | 9 (2) | 8 (5) | 0.1 | 0 | 8 (6) | 0.08 |
| *Prevotella melaninogenica* | 6 (3) | 14 (3) | 14 (9) | 0.4 | 6 (3) | 13 (10) | 0.2 |
| *Peptostreptococcus anaerobius* | 2 (1) | 18 (4) | 3 (2) | 0.006 | 2 (1) | 8 (6) | 0.2 |
| *Clostridialis* BVAB-3 | 0 | 5 (1) | 5 (3) | 0.1 | 2 (1) | 3 (2) | 1.0 |
| *Porphyromonas* | 0 | 5 (1) | 5 (3) | 0.3 | 2 (1) | 4 (3) | 0.6 |
| *Lactobacillus jensenii* | 9 (4) | 0 | 0 | 0.02 | 4 (2) | 3 (2) | 1.0 |

^1^Taxa are given present at >1% relative abundance among at least 3% of the women at baseline.

^2^ Chi-square test

^3^ Present in all samples at a >1% abundance, therefore the proportion of women with a relative abundance higher than the median abundance across all samples is given. The median abundance was 37% for *L. iners* and 19% for *G. vaginalis*.
